# Historical Linguistics of Sign Languages: Progress and Problems

**DOI:** 10.3389/fpsyg.2022.818753

**Published:** 2022-03-09

**Authors:** Justin M. Power

**Affiliations:** Department of Linguistics, University of Texas at Austin, Austin, TX, United States

**Keywords:** historical linguistics, language change, sign language, language relationships, language families

## Abstract

In contrast to scholars and signers in the nineteenth century, William Stokoe conceived of American Sign Language (ASL) as a unique linguistic tradition with roots in nineteenth-century *langue des signes française*, a conception that is apparent in his earliest scholarship on ASL. Stokoe thus contributed to the theoretical foundations upon which the field of sign language historical linguistics would later develop. This review focuses on the development of sign language historical linguistics since Stokoe, including the field's significant progress and the theoretical and methodological problems that it still faces. The review examines the field's development through the lens of two related problems pertaining to how we understand sign language relationships and to our understanding of cognacy, as the term pertains to signs. It is suggested that the theoretical notions underlying these terms do not straightforwardly map onto the historical development of many sign languages. Recent approaches in sign language historical linguistics are highlighted and future directions for research are suggested to address the problems discussed in this review.

## Introduction

Today, signers and scholars alike commonly view each sign language as representing a distinct linguistic tradition. We consider *American* Sign Language to be distinct from *British* Sign Language in part because distinct linguistic conventions have respectively evolved within the American and British signing communities. We also recognize the recent genesis of new traditions, such as the emergence of Nicaraguan Sign Language beginning in the 1970s (Polich, 2005) and of Israeli Sign Language beginning in the 1930s (Meir and Sandler, 2008). According to this contemporary view, a view that developed in significant part due to the ideas of William Stokoe, sign languages have histories.

However, a different view of sign languages, a universalist view, was common in the eighteenth and nineteenth centuries, the period during which many contemporary sign languages emerged in connection with schools for the deaf. Earlier scholars and signers thought of signed language as a universal human language, “the native language of man” (Peet, 1853). For this reason, signed language was commonly called “the language of signs,” without social or geographic modifiers (Baynton, 1996). Insofar as differences were recognized in the signing of geographically distinct communities, the differences were likened to dialectal variation in one common language (Peet, 1853; Baynton, 2002). Thus, Laurent Clerc of France, shortly after arriving in the U.S. in 1816 and upon meeting Alice Cogswell, who would become his first student at the American School for the Deaf, was not surprised to find that he could easily communicate with her in sign: “so true it is, as I have often mentioned before, that the language of signs is universal and as simple as nature” (Clerc, 1852, p. 107).

As a universal language, signed language was not thought to have a historical dimension in the same way that spoken languages were thought to have. Around the mid-nineteenth century, August Schleicher and others had begun to trace the ramification of eight spoken language families from Indo-European (e.g., Schleicher, 1853). In roughly the same period, Thomas H. Gallaudet (1847, p. 56) theorized that the “natural language of signs” had its origins in deaf children's “natural, spontaneous facility.” Gallaudet and his contemporaries held that the similarity of signs among deaf students in schools across Europe and the U.S., and even among Native American signers, had a simple explanation: “[these signs] originate from elements of this sign-language which nature furnishes to man wherever he is found” (Gallaudet, 1847, p. 59). On this view, as the universal, natural expression of all humans, both deaf and hearing, signed language transcended history. When nineteenth-century scholars of signed language, most of whom were professional educators, wrote about history, they most often had in mind the history of pedagogical approaches—with particular focus on manualist vs. oralist methods in deaf education—but not the history of the sign languages that had developed in signing communities (Baynton, 1996; Edwards, 2012).

How has the contemporary view of sign languages as distinct traditions, each with its own unique history, come to differ so markedly from the earlier universalist view? Stokoe's work represents one critical inflection point in the intellectual history of the study of signed language and in the development of the historical linguistics of sign languages. For Stokoe (1960, p. 5) “the natural language of signs” was a “false entity.” In contrast to the views of his universalist predecessors, Stokoe (1960, p. 3) held that a “natural” sign language was “a language system of visual symbols” embedded in a language community, such as “the sign language of the American deaf.” Although historical linguistics was not the primary focus of Stokoe's scholarship, he conceived of ASL as a unique linguistic tradition with roots in nineteenth-century *langue des signes française* (LSF), a conception that is apparent in his earliest scholarship on ASL (Stokoe, 1960; Stokoe et al., 1965). Thus, Stokoe contributed to the theoretical foundation upon which linguists would study not only the history of ASL but also the histories of many other sign languages. Only when sign languages came to be seen as the linguistic traditions of distinct signing communities could broader questions be foregrounded about their histories; and only then could a discipline develop that would focus on the historical linguistics of sign languages.

### Sign Language Historical Linguistics Since Stokoe

Historical linguistics is broadly interested in understanding language change, including how languages change as they diversify from a common ancestral language and how languages change as the speakers and signers of distinct languages come into contact with one another. One principal aspect of the endeavor to understand language change has been the study of language relationships, an area that encompasses the reconstruction of protolanguages, the classification of languages in families, and the subgrouping of more closely-related languages within those families. Following Stokoe, the study of language relationships among sign languages has arguably been the primary area of focus for sign language historical linguists. When sign languages were seen to represent distinct linguistic traditions, the question naturally arose as to how those distinct traditions have developed in relation to one another.

From the 1960s onward, sign scholars have made important advances in our understanding of the histories of many sign languages and of language change in the gestural-visual modality; many of these advances are highlighted in Section Early progress in sign language historical linguistics. Notwithstanding these considerable achievements, I will argue that there remain fundamental theoretical and methodological problems that hinder further progress in sign language historical linguistics. Sign scholars have adopted notions from traditional historical linguistics, such as the language family and cognacy, to theorize the relationships of sign languages and the historical relations of their constituent signs and features. Scholars have also adapted historical comparative methods from that discipline, such as lexicostatistics, to study sign language relationships. The appropriateness of these theories and methods to the historical study of sign languages has, in my view, received insufficient attention to date.

Here I examine the development of sign language historical linguistics since Stokoe through the lens of two related problems pertaining to how we understand sign language relationships and to our understanding of cognacy, as that notion is used to describe the historical relations of signs. I will argue that the relevant theories and methods from historical linguistics do not straightforwardly map onto the historical development of many sign languages. I show how, in light of the problems highlighted here, sign scholars have developed alternative approaches to study the histories of sign languages. While these innovative approaches have provided insights into the histories of many sign languages, I will show that, in some cases, they have also masked important characteristics of sign language change.

In Section Relationships among spoken languages and among signed languages, I examine the first problem, which concerns our theorization of sign language relationships. Do sign languages that are said to be historically related indeed share the same type of relationship that characterizes the spoken languages in a language family (Zeshan, 2006, 2013; Campbell, 2018; Reagan, 2021)? I will show that assumptions underlying theories about spoken language relationships, specifically those pertaining to the intergenerational transmission of language, do not hold for the diversification of sign languages in many sign language families.

The second problem (Section The identification of cognates) concerns our understanding of the cognacy relation among signs. This problem has both a theoretical and a methodological dimension. The theoretical dimension of this problem is related to the first problem mentioned above—the cognacy relation in traditional historical linguistics obtains only among lexical items and linguistic features that have been inherited *via* a specific process of diversification (e.g., Thomason and Kaufman, 1988; Ringe et al., 2002). Where this process has not characterized the diversification of sign languages, the cognacy relation may not be appropriate to describe the historical relations of signs. The methodological problem concerns our ability to identify patterns that uniquely differentiate inherited signs from those that have entered a language *via* processes other than inheritance, such as borrowing. I will show that sign scholars have not yet developed rigorous methods for identifying inherited signs. I argue that, lacking such methods, it is more appropriate to understand the relations of signs among sign languages that are thought to be related as an etymological relation, rather than a cognacy relation.

## Early Progress in Sign Language Historical Linguistics

Stokoe's work on ASL played a pivotal role in the recognition that sign languages indeed have a historical dimension, but progress in understanding the particular histories of these languages, their relationships, and the processes driving historical change in them has been the work of other scholars. These scholars have benefited from the methodological and theoretical developments of the past two centuries in historical linguistics. Early in the development of sign language historical linguistics, lexicostatistics and glottochronology, quantitative approaches that had been developed to study the histories of spoken languages (e.g., Swadesh, 1955; Gudschinsky, 1956), were adapted to the study of sign language data, as was Swadesh's list of basic vocabulary (Woodward, 1978). In interpreting the results of these methods, scholars adopted from historical linguistics the same metaphors to describe language relationships, such as the language family, that had developed in that discipline (see Reagan, 2021 for a recent discussion). They also adopted much of the same terminology that had developed to describe the historical relations of words, such as the relations of cognacy and borrowing, and applied these same notions to the historical relations of signs (Woodward, 1978, 1991). The emergence of many sign languages was theorized in terms that had developed for the analysis of spoken languages. For example, the emergence of ASL was characterized by some scholars as a creolization process with parallels to the emergence of spoken language creoles (Fischer, 1978; Woodward, 1978; Meier, 1984; but see Lupton and Salmons, 1996).

As with the study of language relationships, the study of change in sign languages was influenced by the theories and methods of traditional historical linguistics. Frishberg's (1975) seminal study of historical change in ASL drew connections between the processes driving change in that language and processes such as assimilation and lexicalization in diachronic change in spoken languages. Frishberg argued that other changes, such as the centralization of signs articulated below the neck and the lateralization of signs articulated at the face, reflected tendencies toward articulatory and perceptual ease. Both of these mechanisms of change have parallels in theories of spoken language change (Ohala, 1981, 1993). Battison et al. (1975) adopted the type of variationist approach that had been introduced by Weinreich et al. (1968) to argue that thumb extension in a class of ASL signs—specifically, signs with an extended index finger—represented a historical change in progress in the American deaf community.

By adapting the methods and theories of historical linguistics to the study of sign languages, early scholars made important advances in our understanding of sign language relationships. Woodward (1978) showed that historical relationships among sign languages can be reflected in contemporary linguistic data. Based on quantitative measures of shared cognates, he estimated that ASL and LSF—two languages that were thought to be related on extralinguistic grounds—share 61% of their basic vocabularies. He also studied the intergenerational transmission of ASL signs in the American deaf community, finding that greater than 99% of the signs seen in videos of ASL signers from the early twentieth century have reflexes in contemporary ASL. Thus, Woodward's early work suggested that a historical signal can be identified and measured both in the diversification of sign languages and in the intergenerational transmission of a sign language.

Other studies suggested that related and unrelated sign languages could be reliably differentiated by using the historical comparative methods that had been adapted to study sign languages. In their comparison of British, Australian, and New Zealand Sign Languages, three languages that were hypothesized to be related, McKee and Kennedy (2000) found that between 79 and 87% of these languages' basic vocabularies were cognate—though their operationalization of the term “cognate,” and perhaps also their theory underlying that notion, differed from Woodward's (see Woodward, 2011 for a discussion). In contrast, when these languages were compared with ASL, which was hypothesized to be unrelated to any of the other languages, only between 26 and 32% of their basic vocabularies were found to be, in their terms, cognate. Similarly, in a study of the sources of vocabulary in *Lengua de Señas Mexicana* (LSM), Guerra Currie et al. (2002) found a relatively high percentage of phonologically-similar vocabulary (38%) when comparing that language with LSF, a language that was hypothesized to be related. In contrast, when comparing LSM with *Lengua de Signos Española* and with *Nihon Syuwa* (Japanese Sign Language)—two languages thought to be unrelated to LSM—the percentages of phonologically-similar vocabulary were lower (33 and 23%, respectively).^1^

As in the two studies just mentioned, the methods first used by Woodward to study the relationship of ASL and LSF have been applied to study sign language relationships in other parts of the world. Woodward himself conducted several historical comparative studies of sign languages in Costa Rica, South Asia, Thailand, and Vietnam (Woodward, 1991, 1993, 1996, 2000, 2011). In each of those studies, Woodward used lexicostatistical methods together with quantitative thresholds that were based on expected levels of shared cognates to decide whether the sign varieties that he studied were dialects of the same language. Sign varieties that were inferred to be distinct languages were classified into families. In more recent work, Clark (2017) used a similar approach to study sign varieties in Peru, finding that there are two distinct sign languages in use in that country and that a third variety is a hybridized variety with elements of the two distinct languages. Other scholars have adapted Woodward's methods to argue for the historical relationships, inter alia, of sign languages in Eastern Europe, such as *Russkii Zhestovyi Yazyk* and *Ukrayinska Zhestova Mova* (Russian and Ukrainian Sign Languages; Bickford, 2005), of *Nihon Syuwa* and *Táiwān Shǒuyǔ* (Japanese and Taiwan Sign Languages; Sasaki, 2007), and of sign languages in the Middle East, such as *Lughat il-Ishaarah il-Urduniah* and *Lughat al-Ishārāt al-Filisṭ*&#*x00131;̄niyyah* (Jordanian and Palestinian Sign Languages; Al-Fityani and Padden, 2010).

In addition to the advances in our understanding of the historical relationships among sign languages and in the methods for studying those relationships, sign scholars have also made progress in understanding language change in the gestural-visual modality. Radutzky's (1989) study of historical change in *Lingua dei Segni Italiana* (LIS) investigated the categories of change that had been identified in Frishberg (1975). She found that many of the same diachronic changes that Frishberg had described for ASL, such as changes in the shape of the nondominant hand and in the lateralization of signs articulated at the face, had also occurred in LIS. She also found that one diachronic change in LIS paralleled the type of change in thumb extension that had been identified by Battison et al. (1975) as an ongoing change in the American deaf community. As with Frishberg's account of diachronic changes in ASL, Radutzky identified articulatory and perceptual ease as important drivers of change in LIS.

Like Frishberg, Supalla and Clark (2015) investigated historical sources—particularly, video recordings of ASL signers in the early twentieth century—to understand the origins of lexical signs and grammatical constructions in ASL. Their analysis of video recordings in addition to more static sources, such as historical dictionaries (see Frishberg's analysis of Long, 1918), allowed them to observe historical signs in a range of phrase- and discourse-level contexts. They observed that many signs in contemporary ASL have developed from historical compounds and collocations, which have undergone diachronic processes of reduction and semantic shift. They argued that many changes affecting forms in the ASL of the early twentieth century had been driven by grammaticalization processes (see Hopper and Traugott, 1993).

Just as Battison et al. (1975) used a variationist approach to study changes in handshapes, later scholars took a similar approach to investigate changes in the locations of signs. Lucas et al. (2001) examined a class of signs in ASL that is defined phonetically by articulation at the forehead or temple in citation form. They found a positive correlation between the height of signers' articulations and their ages: older signers produced more tokens at higher locations on the head, while middle-aged and younger signers produced more tokens with lower articulations. The authors tentatively concluded that the differences in location in this class of signs represented a change in progress in the American deaf community. In a study examining a similar class of signs in Australian and New Zealand Sign Languages, Schembri et al. (2009) also found that sign articulations were positively correlated with signers' ages. In both studies, the authors hypothesized that the mechanism driving the diachronic changes was articulatory ease, since higher articulations presumably require more effort compared to lower articulations (Mauk, 2003; Napoli et al., 2014).

In sum, the preceding brief survey of sign language historical linguistics has highlighted two critical areas of progress since Stokoe. First, scholars have developed methods that can identify historical signal in contemporary sign data; that historical signal is sufficiently robust to differentiate sign languages that are thought to be related on extralinguistic grounds from those that are thought to be unrelated. Second, real time and apparent time studies of change in sign languages have identified diachronic changes that have occurred in more than one sign language. These discoveries suggest that the field might eventually identify a comprehensive set of common diachronic changes that occur in languages in the gestural-visual modality. The changes that have already been identified have been argued to be driven by mechanisms, such as ease of articulation and perception, that have also driven many changes in spoken languages.

## Relationships Among Spoken Languages and Among Signed Languages

What does it mean to say that languages are *related*? The word *related* has multiple senses; one of these senses means “connected or having relation to something else” (Oxford English Dictionary, 2021b). Thus, one answer to the initial question could be that two languages are related—that is, connected—if they share words or linguistic features. This view of language relationships would be unconcerned with how shared words and features have entered languages; instead, the main consideration on this view would be how closely connected, or perhaps how similar, the two languages are at a particular point in time, given some metric of connection. Hence two previously unrelated languages could become related, if, for example, the speakers of these languages begin to borrow words from one another; conversely, two related languages could become unrelated, if speakers would cease to use the words or features they once had in common. Because the connections among languages and their similarity may change over time, so too, on this view, might language relationships change.

The term *related* in traditional historical linguistics differs from the view just described. When deciding whether two languages are related, historical linguists are not concerned with their similarity or with connections among their speakers *per se*, but rather with the processes that have resulted in these languages' shared words and features. Language relationships in historical linguistics are theorized in a way that parallels the evolutionary relationships of organisms (Atkinson and Gray, 2005). For example, birds and bats share many morphological similarities; yet from an evolutionary perspective, bats are more closely related to humans than they are to birds because bats and humans share a more recent common ancestor (Morrison et al., 2015).

In historical linguistics, common ancestry has been fundamental to the meaning of language relationships. Just as offspring inherit DNA from an ancestor, a younger generation of speakers is thought to acquire, or inherit, a language system from an older generation, including that language's words and features. For example, the Proto-West-Germanic verb ^*^laidijan “lead” (Ringe and Taylor, 2014) is thought to have been inherited by successive generations of children along a chain of language transmission events down to the present day. As Proto-West-Germanic diversified, ^*^laidijan came to be inherited in distinct speech communities, in which the word subsequently underwent distinct sound changes, resulting in, for example, Old English (*lædan*), Old Dutch (*leiden*), and Old High German (*leiten*) (Ringe and Taylor, 2014). Although the contemporary reflexes of these words—namely, English *lead*, Dutch *leiden*, and German *leiten*—differ in their phonological forms, they have all been inherited along chains of language transmission events that trace back to a common ancestor, namely, Proto-West-Germanic.

In contrast to the process of inheritance, consider the process of borrowing, which represents a different pathway by which words and linguistic features may enter a language. During the 1940s, adult speakers of American English, initially soldiers, evidently borrowed the word ‘honcho', meaning leader or person in charge, from Japanese *hancho* [*han* “corps, squad” and *cho* “head, chief”; (Online Etymological Dictionary, 2021; Oxford English Dictionary, 2021a)]. Although in this case a word with etymological origins in Japanese entered into American English, and although American English and Japanese have become, in a sense, more closely connected after this borrowing event, historical linguists would not say that the two languages are related because of the borrowing event. The American English word *honcho* and the Japanese word *hancho* were not intergenerationally inherited from a common ancestral language as constituents of that language system.

### *Genetic* Language Relationships

Characteristics of the language transmission process itself play a fundamental role in how language relationships are understood in historical linguistics.^2^ These characteristics are not fixed; instead, language transmission is sensitive to social and cultural variation. The network of social connections through which language transmission occurs can differ. For example, language can be transmitted from parent to child, from nonparental adult to child, and among peers; and many children are exposed to multiple languages along these various pathways (Cavalli-Sforza and Feldman, 1981; Mufwene, 2008). The typical settings within which language is transmitted can also differ. For example, in some speech communities, language may be primarily transmitted to children in the home, at least early on. Sign languages too may be primarily transmitted to children in the home in some village signing communities (Zeshan and de Vos, 2012), in multi-generational family signing communities (Dikyuva, 2012), in relatively small networks of families with deaf members (Hou, 2016), and, in general, in any setting in which older generations sign with younger generations (Newport and Meier, 1985; van den Bogaerde and Baker, 2016; German, 2021). But, in some signing communities, an important setting for language transmission to children has been the deaf school and dormitory (Singleton and Meier, 2021).

Language transmission can also occur at differing ages and hence at differing stages of cognitive development. Power and Meier (2021) report that there were few young children at the American School for the Deaf in Hartford during the school's first 50 years because its minimum age for admission was 8 years old or higher. Less than 1% of 1,700 students were under age 8 at enrollment during that period, and the average age at enrollment was 14.4 years old (*SD* = 5.2 years). The school's admission policy likely caused many deaf children, particularly those without access to visual language at home, to experience language deprivation in childhood, an experience which can have negative consequences for language acquisition. The age at which an individual acquires a sign language has been shown to affect language processing (Morford, 2003), second language acquisition (Mayberry et al., 2002), as well as the acquisition of verbs and basic word order in ASL (Newport, 1988; Cheng and Mayberry, 2020). When late learners transmit language to a subsequent generation, the language system itself may have varying levels of complexity and consistency (Senghas and Coppola, 2001; Singleton and Newport, 2004). In sum, it seems that many aspects of the language transmission scenario can vary—including characteristics of the transmitter, transmission pathway, language, setting, and acquirer.

Among the overall set of potential language transmission scenarios, one scenario has been termed *normal*, or typical, because it arguably occurs under typical social conditions.^3^ According to Thomason and Kaufman (1988, p. 9–10), normal transmission occurs “from parent generation to child generation and/or *via* peer group from immediately older to immediately younger, with relatively small degrees of change over the short run, given a reasonably stable sociolinguistic context.” When successive generations inherit a language *via* this type of transmission, the process results in a chain of languages, each one having been derived from the immediately preceding language. Ringe et al. (2002, p. 63) refer to this process as *linguistic descent*, which they define in the following way: “A language (or dialect) Y at a given time is said to be descended from language (or dialect) X of an earlier time if and only if X developed into Y by an unbroken sequence of instances of native-language acquisition by children.” The process parallels asexual biological reproduction in that each derived language is thought to have just one antecedent language.

The notions of normal transmission and linguistic descent have been critical to the understanding of language relationships in historical linguistics. Languages are related and belong to the same language family if they are derived *via* linguistic descent from a common ancestral language (Thomason and Kaufman, 1988; Thomason, 2002; but see DeGraff, 2001; Mufwene, 2003 for critiques). In the terminology of many historical linguists, languages that are related in the way just described are said to share a specifically *genetic* relationship.

### *Nongenetic* Language Relationships?

How do we characterize the relationships of languages that are not derived from a common ancestral language *via* linguistic descent? Historical linguists consider many languages, such as English and Japanese, to be unrelated because no plausible common ancestral language has yet been reconstructed for them; and perhaps none can be, if no such ancestral language existed. English and Japanese have genetic relationships to other languages—just not to each other. Language isolates, such as Basque and Ainu, are also thought to lack genetic relationships to any existing or extant languages (Campbell, 2013). However, language isolates may once have had genetic relationships to some language or group of languages that are now extinct. And, importantly, an isolate is presumably linked *via* linguistic descent to antecedent stages in its own historical development.

What happens if the chain of linguistic descent is broken in a language's historical development—as the development of a creole language has been thought to entail? What if the intergenerational transmission of language differs from the type of transmission described above? How do we characterize the relationships of languages that have not developed *via* linguistic descent? According to Thomason and Kaufman (1988, p. 10), if the chain of linguistic descent is broken at any point, the relationship between languages on either side of the break is not genetic: “the label ‘genetic relationship' does not properly apply when transmission is imperfect.” In addition, as we have seen, in linguistic descent exactly one ancestral language develops into a derived language. Thus, any language that is descended from more than one ancestral language—as creole languages and many sign languages are thought to be—has no genetic relationships to any antecedent language or to any other languages that have descended from those antecedents. These languages with multiple sources “[have] followed a nongenetic pathway of development” (Thomason and Kaufman, 1988, p. 8).

Scholars of creole languages have debated how to characterize the relationships of a creole to its lexifier and its substrates (DeGraff, 2001; Thomason, 2002; Mufwene, 2003). How does a creole's history connect with the histories of the languages that have, at least in part, formed the basis of its lexicon and grammar? If linguistic descent is taken to be definitional in the theory of language relationships, a creole has no genetic relationship to its antecedents because its linguistic system has multiple sources. For example, Thomason and Kaufman (1988, p. 11) contend that mixed languages “by definition...are unrelated genetically to the source(s) of any of their multiple components”; and, similarly, that “a claim of genetic relationship entails systematic correspondences in all parts of the language because this is what results from normal transmission: what is transmitted is an entire language.” Thus, on this view, any language with heterogeneous sources has no genetic relationships to its antecedents or to the contemporary languages that have descended from those antecedents. While these relationships are not considered genetic, the theory does not make clear how to positively define the relationship; witness the unwieldy term “genetic nonrelatedness” in Thomason (2002, p. 105).

### The Diversification of Sign Languages *via* Processes Other Than Linguistic Descent

Sign languages have been grouped into language families based on a variety of types of evidence, including extra-linguistic evidence, such as historical connections among deaf educators and educational institutions, linguistic evidence, and a combination of both types of evidence (see Fischer, 2015; Reagan, 2021 for recent discussions). For example, contemporary ASL and LSF are typically classified together with other sign languages that have some historical connection to the variety or varieties of LSF used in eighteenth- and nineteenth-century schools for the deaf in France, including European sign languages such as *Nederlandse Gebarentaal, Teanga Chomhartha*í*ochta na hÉireann*, and *Lingua dei Segni Italiana* (Sign Language of the Netherlands, Irish Sign Language, and Italian Sign Language) and sign languages of Latin America such as *Lengua de Señas Mexicana* and *L*í*ngua Brasileira de Sinais* (Anderson, 1979; Quer et al., 2010; Abner et al., 2020; Power et al., 2020). Other proposed sign language families include, inter alia, the family of British, Australian, and New Zealand Sign Languages (McKee and Kennedy, 2000), the family including *svenskt teckenspråk* and *L*í*ngua gestual portuguesa* (Swedish and Portuguese Sign Languages; Bergman and Engberg-Pedersen, 2010), and the family including *Nihon Syuwa, Táiwān Shǒuyǔ*, and *Hanguk Sueo* (Japanese, Taiwan, and Korean Sign Languages; Sasaki, 2007).

However, many languages in the sign language families that have been proposed to date evidently are not derived *via* linguistic descent from a common ancestral language because, (i) as with creole languages, many sign languages are thought to have multiple sources; and (ii) the diversification of these languages implicated a break in their intergenerational transmission. First, some scholars have characterized the emergence of ASL as the creolization of LSF with the indigenous sign varieties of nineteenth-century American deaf signers (Woodward, 1978; Groce, 1985). Fischer (1978, p. 329) hypothesized that ASL has been “recreolized” by deaf children in each generation since the early nineteenth century (see also Meier, 1984) because most deaf children do not acquire ASL from birth—roughly 90% do not (Mitchell and Karchmer, 2004). Guerra Currie (1999) speculates that *Lengua de Señas Mexicana* may have emerged in a similar way—that is, the indigenous sign varieties of Mexican deaf signers may have creolized with LSF in the emergence of *Lengua de Señas Mexicana*. The emigration to Israel of Jewish deaf people from a variety of countries in the first half of the twentieth century is thought to have played an important role in the diversification of that language from *Deutsche Gebärdensprache* (German Sign Language) and other sources (Meir and Sandler, 2008). Insofar as the emergence of these and other sign languages have implicated multiple sources, they may not be genetically related to each other in the way that the languages in a spoken language family have been thought to be.

Second, many of the historical relationships among sign languages that are thought to have resulted from connections among deaf institutions and the travels of deaf educators have not been characterized by linguistic descent. For example, the historical relationship between ASL and LSF is understood to be based in large part on the transmission of LSF by Laurent Clerc, a deaf educator who moved from Paris to the U.S. in 1816 in order to teach at the American School for the Deaf in Hartford (Edwards, 2012). Clerc himself had acquired LSF at the age of 12, when he moved to Paris from La Balme to attend the Paris National Institute (Lane, 1984). Arguably, Clerc's acquisition of LSF does not straightforwardly map onto the type of intergenerational transmission said to define genetic spoken language relationships because he did not acquire that language as a child. Additionally, as we have seen, some 99% of Clerc's students in Hartford during the school's first 50 years were above the age of 8 at the time of their enrollment; and the average student enrolled in adolescence (Power and Meier, 2021). Thus, both Clerc's acquisition of LSF and his transmission of that language to his American students arguably were not characteristic of linguistic descent, in the sense under discussion here. Instead, the diversification of ASL from a nineteenth-century variety of LSF evidently entailed a break in the intergenerational transmission of that language.

As with the early development of ASL, the diversification of many other sign languages may not have occurred *via* linguistic descent. For example, another French deaf educator, Édouard Huet, who had apparently acquired LSF at age 12, established schools for the deaf in Brazil (est. 1857) and Mexico (est. 1867; Guerra Currie, 1999). The sign languages that later developed in those countries—namely, *L*í*ngua Brasileira de Sinais* and *Lengua de Señas Mexicana*—have been thought to be historically related to LSF (Quinto-Pozos, 2008). However, while Huet may have driven the establishment of the schools in Brazil and Mexico, Ramsey and Quinto-Pozos (2010, p. 49–50) speculate that, in Brazil, Huet's LSF-origin signs may have “mixed with the varieties of signing that Brazilian Deaf students brought to the school”; and, regarding Mexico, the authors report that “neither sign-medium instruction nor Deaf teachers played a major role in the school” following its establishment.

A deaf Norwegian, Andreas Christian Møller, began attending the school for the deaf in Copenhagen at age 16; he later returned to Norway and established the first school for the deaf in that country in Trondheim (Greftegreff et al., 2015). *Norsk tegnspråk* and *Dansk tegnsprog* (Norwegian and Danish Sign Languages) have been thought to be historically related (Schröder, 1993). In sum, the diversification of *L*í*ngua Brasileira de* Sinais and *Lengua de Señas Mexicana* from a nineteenth-century variety of LSF and of *Norsk tegnspråk* from a nineteenth-century variety of *Dansk tegnsprog* evidently occurred *via* transmission from late learners of those languages.

While Huet and Møller were themselves deaf, the diversification of many other sign languages has occurred in part *via* hearing educators, who were not likely native users of those languages. For example, a hearing priest, Father Tomaso Silvestri, received training in sign language and in pedagogical methods at the Paris National Institute before founding the first public school for the deaf in Italy in 1784 (Quer et al., 2010). A hearing educator of the deaf from Sweden, Per Aron Borg, helped to establish a school for the deaf in Portugal (est. 1823–1828), in which he introduced aspects of *svenskt teckenspråk* to his Portuguese deaf students (Bergman and Engberg-Pedersen, 2010). A hearing teacher, Dorcas Mitchell, introduced a variety of British Sign Language to deaf students in New Zealand in 1868 (Schembri et al., 2010).^4^ Hearing Irish nuns, after learning a variety of LSF during a visit to a school in Normandy, introduced that variety in a school for female deaf students in Dublin; later, the nuns shared their variety with hearing teachers at another school for male deaf students in Dublin (LeMaster and Dwyer, 1991). A variety of BSL was introduced by a hearing teacher and her two deaf children, who had moved from England to establish the first school for the deaf in Uganda (Lule and Wallin, 2010; Lutallo-Kiingi and De Clerck, 2015). The origins of *Ishorai Tojiki* (Tajik Sign Language) have been linked to the introduction of a second language variety of *Russkii Zhestovyi Yazyk* (Russian Sign Language) by a group of hearing educators in the former Soviet Union, who established a school for the deaf in Tajikistan around the 1940s (Power, 2020). In each of these cases, and in many other cases around the world like them, sign languages have been classified in the same language family, even though their diversification has not occurred *via* successive instances of the native acquisition of language by children—that is, not *via* linguistic descent.

Not all sign languages have diversified in close connection with educational institutions in the ways just described. For example, the diversification of Australian Sign Language may have initially occurred *via* the migration of at least one signer of British Sign Language, John Carmichael, who had attended the school for the deaf in Edinburgh (Schembri et al., 2010). Other British Sign Language users immigrated to Australia soon after, such as Carmichael's schoolmate, Thomas Pattison, who would later establish the first school for the deaf in Australia in 1860 (Schembri et al., 2010). Carmichael had five children, at least one of whom, Edward Feeney Carmichael, was deaf (Eaton, 2015). Thus, following their presumptive native acquisition of British Sign Language from their father, Carmichael's hearing children and his deaf son may have played a role in the diversification of Australian Sign Language from the British Sign Language of the nineteenth century *via* linguistic descent. However, Carmichael himself apparently began attending the Edinburgh school at age 9 (Eaton, 2015); similarly, Pattison may have begun his studies there at around age 8 (Cooper, 2014). Prior to their attendance at the Edinburgh school, it is unclear whether either of these individuals had had any exposure to British Sign Language. When viewed through the lens of the theory of genetic language relationships, these signers' relatively late acquisition of British Sign Language may have resulted in a break in the type of chain of child language acquisition events that has been thought to characterize linguistic descent (Ringe et al., 2002; see Section *Genetic* language relationships).

In sum, the relationships of sign languages in many sign language families arguably differ from the types of relationships that are thought to characterize spoken language families because, in many cases, the diversification of languages in these sign families has not occurred *via* linguistic descent. The diversification of many sign languages from antecedent sign languages—such as the diversification of ASL from LSF—may more closely resemble the process described by Mufwene (2009) as “indigenization.” In the context of the diversification of world Englishes from varieties of British English, Mufwene (2009, p. 353) defines linguistic indigenization as a “process whereby a language is adapted to the communicative habits and needs of its (new) speakers in a novel ecology.” In the case of the diversification of ASL from LSF in the early nineteenth century, the novel ecology into which LSF was introduced—initially, New England—certainly differed in numerous ways from the ecology within which LSF had developed to that point. In its adaptation to the American linguistic ecology, with its complex array of novel demographic, social, cultural, and linguistic features, LSF likely changed in profound and complex ways.

### Linguistic Descent and Its Consequences for Theories of Sign Language Relationships

As we have seen, historical connections among sign languages can be reflected in their contemporary forms; for example, sign languages in the French family share similar vocabulary (Woodward, 1978; Guerra Currie et al., 2002), similar structural features (Abner et al., 2020), and similar fingerspelling alphabets (Power et al., 2020). If we accept a historical explanation for many of these similarities, what is the theoretical significance of whether these shared signs and features have been inherited *via* linguistic descent or *via* some theoretically nongenetic pathway?

Linguistic descent crucially implicates the native acquisition of language by children. Labov (2007) argues that differences in the ways that children vs. adults acquire language underlie differences between internal language change and change due to contact. Linguistic descent produces gradual changes (“incrementation”) in a language from generation to generation: “the continuity of dialects and languages across time is the result of the ability of children to replicate faithfully the form of the older generation's language, in all of its structural detail” (Labov, 2007, p. 346). In contrast, “adults do not learn and reproduce linguistic forms, rules, and constraints with the accuracy and speed that children display” (Labov, 2007, p. 349). Thus, if the chain of child language acquisition events is broken, relatively abrupt, chaotic changes may be introduced in the historical development of a language. The diversification of many sign languages has arguably been characterized by abrupt changes of this type. Following diversification, however, the transmission of language in a signing community—for example, of ASL in the American deaf community after the introduction of LSF in the early nineteenth century—could be characterized by linguistic descent.

That the diversification of many sign languages has arguably been characterized by abrupt changes introduces a second set of problems. In traditional historical linguistics, methods for identifying inherited words—that is, cognates—among related languages rely on the type of gradual and regular changes that Labov has argued are characteristic of linguistic descent. If, for example, the diversification of sign languages in the French family has implicated abrupt changes, and not gradual, regular changes, then it may not be possible to use the methods of traditional historical linguistics to identify historically-related signs among languages in a sign language family. Before considering this second set of problems for sign language historical linguistics, I first raise a number of critiques of the theory of genetic language relationships.

### Critiques of the Theory of Genetic Language Relationships

To this point, I have attempted to bring into stark relief one aspect of the problem situation confronting sign language historical linguistics. The theoretical dimension of this problem relates to the key roles played by the notions of normal transmission and of linguistic descent in the theory of genetic language relationships. My aim has been to emphasize that these notions should not be uncritically adopted in theorizations of the historical development of sign languages and of their relationships to each other. In this section, I turn the focus onto the theory of genetic language relationships by raising two critiques; see Mufwene (2003, 2008) and DeGraff (2001) for additional critiques from the field of creole studies.

The first critique arises through a comparison of the theory of genetic language relationships with the putatively parallel theory in evolutionary biology. Although these theories share many similarities, the underlying processes of linguistic and biological evolution nevertheless fundamentally differ (Atkinson and Gray, 2005). Hence it may be misleading to use terminology such as genetic and nongenetic in theories of language relationships. In the theory of genetic language relationships, as we have seen, some pathways of development are considered nongenetic; however, there are no nongenetic pathways of development in evolutionary biology. Every life form has inherited genetic material from at least one antecedent, and hence every species—arguably, the notion that most closely parallels the notion of a language in the current discussion (Mufwene, 2008)—has developed *via* fundamentally genetic pathways. Relatedly, all species are represented on the one evolutionary tree of life; hence all species have genetic relationships (Maddison et al., 2007). Thus, if the theory of genetic language relationships adopts terminology such as genetic from evolutionary biology, why does the theory allow for some languages to lack relationships?

Furthermore, because creoles, mixed languages, and many sign languages are thought to have multiple antecedents, their development, according to the theory of genetic language relationships, has been nongenetic (Thomason and Kaufman, 1988; Ringe et al., 2002). That is, linguistic descent implicates asexual reproduction, in a sense; whereas the parallel to sexual reproduction, or perhaps to hybridization, in language formation is considered a nongenetic pathway of development. In contrast to this aspect of the theory of genetic language relationships, both sexual reproduction and hybridization in biology are fundamentally genetic processes. In sum, if intergenerational language transmission and language relationships are theorized in such starkly different ways compared with biological evolution and evolutionary relationships among species, then perhaps terms such as genetic and nongenetic are not appropriate in theories of language relationships.

There is at least one apparent limitation to the theory of genetic language relationships that pertains to this first critique. If some languages have developed along nongenetic pathways, then how does one describe the historical relations of vocabulary and linguistic features that apparently have shared common pathways of historical development? For example, Fischer (1996) has argued that the sign in ASL representing the number three has its origins in nineteenth-century LSF. But, if ASL has not developed from the LSF of the nineteenth century *via* linguistic descent—or indeed from any other language by that process—how do we describe the historical relation obtaining between the contemporary signs in ASL and LSF for the number three? See Section The identification of cognates for a discussion of this problem as it relates to the term cognate.

In one sense, the theory of genetic language relationships divides languages into two classes: one class of languages has genetic relationships because these languages have developed *via* linguistic descent; whereas languages in the other class have no genetic relationships due to characteristics of their intergenerational transmission. The traditional methods in historical linguistics for studying language relationships only properly apply to the former group of languages. For instance, scholars applying the Comparative Method presume that the languages being compared are related (Nichols, 1996; Hale, 2015). How does one study relationships among languages that have developed, according to the theory, along nongenetic pathways?

The second critique pertains to the notion of normal transmission and the emphasis in that notion on the native acquisition of language by children. Because most deaf children are born into hearing, non-signing families (roughly 90%, Mitchell and Karchmer, 2004), these children often experience delays in their exposure to visually-accessible language. Hence the typical situation for language transmission, when considering many signing communities, is not the type of parent-to-child, intergenerational transmission that is assumed to be normal in the notion of normal transmission described above. Costello et al. (2008) note that, in smaller signing communities, such as the community in Basque Country, there may be extremely few deaf signers who could be considered native signers, given the notion of native that is assumed in speech communities. These authors suggest that the number of signers who have acquired their community's language from birth may depend on factors such as the community's marriage patterns and the prevalence of genetic deafness in the community. Because these factors likely vary across language communities, the patterns of typical language transmission in these communities may vary as well.

Cheng et al. (2021) suggest that the terms “native speaker” and “native signer” have sometimes been used by scholars in ways that conflate differing aspects of language acquisition, proficiency, and identity. In light of differences in the demographics of many signing vs. speech communities and, relatedly, in light of differences in the typical pathways of language transmission in these communities, the authors recommend that scholars carefully disentangle the various assumptions that constitute the category of native speaker or native signer. Arguably, a more nuanced theorization of linguistic experience and language transmission would allow historical linguists to more fully capture the natural complexity in how languages change and in how they are related to one another. In sum, we might expect of a theory of language relationships that it engages with the complex patterns of intergenerational language transmission and of language diversification that actually occur in the world.

## The Identification of Cognates

At the beginning of Section Relationships among spoken languages and among signed languages, I contrasted the inheritance of ^*^laidijan “lead” from Proto-West-Germanic in contemporary English, Dutch, and German with the borrowing of *honcho* from Japanese by American English speakers. Because English *lead*, Dutch *leiden*, and German *leiten* have been inherited *via* linguistic descent from Proto-West-Germanic, the contemporary words are said to be cognates. Trask (2000, p. 62) defines the term cognate as “one of two or more words or morphemes which are directly descended from a single ancestral form in the single common ancestor of the languages in which the words or morphemes are found, with no borrowing.” Because cognates are inherited *via* linguistic descent, by comparing them across related languages linguists may discover information about the internal structure of a language family—that is, the sequence of language diversification events in that language family. In contrast, borrowings do not provide the same type of historical information—certainly, not at the point of the borrowing event—because they were not inherited *via* linguistic descent as constituents of a common ancestral language.

In traditional historical linguistics, the Comparative Method has been the principal methodology used to identify cognates among related languages. Even in more recent quantitative approaches in historical linguistics, the data have typically comprised cognates that had been previously identified using the Comparative Method (e.g., Gray and Atkinson, 2003; Kolipakam et al., 2018). The Comparative Method depends on the assumption that sound change can be regular (Rankin, 2003; Hale, 2015). The methodology seeks to identify regular sound correspondences across semantically similar words; see Campbell (2013) for a comprehensive discussion of the methodology. In the example above, the correspondence in the second consonants across English (-d), Dutch (-d-), and German (-t-) regularly recurs in many other words in those languages (e.g., in English *ride*, Dutch *reijden*, and German *reiten*). The most parsimonious explanation for this regular correspondence is the genetic hypothesis (Hockett, 1965)—that is, that the contemporary words have been inherited from a common ancestral language.

We do not yet know if sign change can be regular in the way that sound change in spoken languages has been argued to be (Labov, 2020). None of the diachronic changes identified among sign languages have yet been shown to occur uniformly, given a defined phonetic context (Power et al., 2019); nor have regularly recurring correspondences of the type described above been identified across sign languages that are thought to be related. In the previous section, I highlighted a potential explanation for this apparent lack of regular correspondences: namely, the diversification of many sign languages in sign language families may not have been characterized by linguistic descent. Hence we would not expect to find regular correspondences across these sign languages because regular correspondences result from the type of gradual change that is characteristic of linguistic descent.

If sign change cannot be regular, or if the historical development of many sign languages has not resulted in regular correspondences across languages that are thought to be related, then it is not possible to use the Comparative Method to identify cognate signs. Because the Comparative Method in traditional historical linguistics is so tightly intertwined with the identification of cognates through regular correspondences, it is unclear how cognates ought to be identified among sign languages that do not exhibit such correspondences—or, indeed, whether signs that are apparently historically-related, given some alternative method to identify such signs, should be considered cognates.

One further feature of all known sign languages that complicates the identification of cognates is the apparently greater prevalence of iconic and indexical representations in the lexicons of signed vs. spoken languages (Perniss et al., 2010). As a matter of course, historical linguists of spoken languages avoid iconic, or onomatopoetic, vocabulary in their historical comparisons because phonological similarities, and even apparent correspondences, among such vocabulary may not reflect shared history (Campbell, 2013; but see Joseph, 1987). The avoidance of iconic vocabulary in historical comparisons of spoken languages developed from the work of early theorists, such as Meillet (1925/1967, p. 14), who stressed that our ability to make historical inferences based on language depends on the conventional, but not “natural,” connection between form and meaning: “If the meaning to be expressed by language were linked by a natural connection, loose or strict, to the sounds which indicate it, that is, if by its own value, apart from tradition, the linguistic sign evoked an idea in any way… all linguistic history would be impossible.” As we have seen, however, sign scholars such as Woodward have developed methods that apparently identify historical signal in comparisons of sign language vocabulary—despite the high prevalence of iconic representations. Nevertheless, in agreement with Meillet, the historical signal that the methods of sign language historical linguistics apparently identify is, in a sense, fuzzy. That is, when comparing a set of putatively cognate signs across sign languages, no currently-available methodology rigorously differentiates signs that are similar due to iconicity from those that have been inherited from a common ancestral language.

In the next section, I describe how, absent regular correspondences, sign scholars have adapted their theories and methods to confront the problem of identifying historically-related signs.

### Theoretical Adaptation of the Cognate

The inability to identify regular correspondences using the Comparative Method has, in my view, significantly shaped the field of sign language historical linguistics. Sign scholars have developed alternative theories and inferential frameworks for understanding the historical relations of sign vocabulary and, relatedly, the historical relationships of the languages themselves. These alternative approaches fundamentally differ from the Comparative Method because they do not rigorously identify vocabulary that has been inherited from a common ancestor or differentiate that vocabulary from borrowings. Here I briefly highlight two approaches in which the notion of cognacy has been expanded to encompass both inherited vocabulary and borrowings. In the next section, I describe two classes of methods that sign scholars have developed as alternatives to the Comparative Method.

The first approach was developed by James Woodward, who has argued for an adaptation of lexicostatistical methods that allows sign scholars to classify sign languages into families without identifying specifically inherited vocabulary.

“A particular advantage to lexicostatistics that is not shared by the comparative method is that lexicostatistics does not assume that languages in the same language family necessarily came from one common ancestor—merely that something has influenced these languages so that they have become similar to each other. This something could be a common ancestor, or it could be extensive borrowing, hybridization, and/or creolization” (Woodward, 2011, p. 41).

In Woodward's approach, the sign language family differs from the spoken language family because it is based on *influence* rather than inheritance. Influence is conceived as a broad category encompassing both inherited and borrowed features. In addition, lexicostatistics is seen by Woodward to be tightly intertwined with the aims of sign language historical linguistics in general, taking the place of the Comparative Method in traditional historical linguistics.

A second alternative approach is found in Supalla and Clark's (2015) notion of “sign language archaeology” (see also Shaw and Delaporte, 2014). Their archaeological, or perhaps philological, approach deals mainly with historical texts, videos, and descriptions of sign meanings and their origins. As with Woodward's approach, these authors take an expansive view of cognacy: “[t]o determine a cognate relationship, researchers make an informed decision with the help of either folk etymology or additional scientific excavation for evidence of historical relatedness between the current LSF form and the modern ASL form” (Supalla and Clark 2015, p. 90). The archaeological approach to identifying cognates does not seek to differentiate vocabulary in ASL that has been inherited *via* linguistic descent from vocabulary that has entered the language *via* other processes.

Supalla and Clark (2015, p. 190) also point out that folk etymologies about the origins of signs—and hence their potential cognacy relations to other signs—“arise when there is a gap in knowledge about the true history of a word”; typically, these etymologies “are not substantiated by history or fact.” Over time, according to these authors, folk etymologies may come to constitute shared cultural knowledge that is “transmitted across generations as part of sign language culture.” Thus, folk etymologies may simultaneously represent important cultural knowledge that nevertheless may not provide an accurate description of the historical development of a sign.

In Section Early progress in sign language historical linguistics, I highlighted several of the important contributions that sign scholars, including the scholars discussed above, have made to our understanding of the histories of many sign languages and of language change in the gestural-visual modality. Many of these contributions have been due to these scholars' innovative approaches in the face of the theoretical and methodological problems that I have described here. However, these innovations have also created new issues. The theoretical adaptation of the term cognate has avoided the methodological problem raised above because, in this adapted view of the cognate, inherited signs are not differentiated from borrowings. However, while sign scholars have often used the term cognate to describe historically-related signs (but see Guerra Currie et al., 2002), it is important to recognize that this notion in sign language historical linguistics differs from the notion of the cognate in traditional historical linguistics. Consequently, sign language relationships that are based on this expanded notion of the cognate theoretically differ from relationships among spoken languages, which are strictly based on inheritance.

### Methodological Adaptations for Identifying Historically-Related Signs

Sign scholars have developed two main approaches for making inferences about the historical relations of signs. In contrast to the aims of the Comparative Method, these approaches have been concerned with identifying historically-related vocabulary—potentially including both inherited and borrowed signs. The first approach adjusts the parameters of the Comparative Method such that correspondences are not required to regularly recur; this approach also incorporates an implicit model of how signs may historically change. The second approach uses measures of phonetic similarity to make inferences about the historical relations of signs; this approach does not include a model of historical change. The strength of the first approach is that it incorporates a theory of diachronic sign change in the historical inference procedure. The second approach includes a clearer inferential procedure, which, to some extent, mitigates the potential for systematic bias present in the first approach.

#### Woodward's Approach to Identifying Cognates Without Regular Correspondences

In perhaps the earliest work applying methods from historical linguistics to study the histories of sign languages, Woodward (1978) adapted lexicostatistical and glottochronological methods in a lexical comparison of ASL and LSF. He used Swadesh's 200-word list of basic vocabulary as the basis for comparing the two languages; he also used Gudschinsky's (1956) methodology for making cognate inferences.

The appeal of Gudschinsky's methodology may have come from its use of the notion of *probable cognacy*, which in effect loosened the requirement of the Comparative Method that correspondences regularly recur. For example, her “criterion c” allows sounds that differ across potential cognates to be analyzed as “agreeing” (i.e., corresponding) if the sounds' environments might plausibly have conditioned their difference—even if, crucially, the correspondence does not regularly recur in other words (Gudschinsky, 1956, p. 184). The methodology is less rigorous compared to a procedure that requires correspondences to regularly recur in other vocabulary, given the same conditions. Like Starostin's (2013) “preliminary lexicostatistics,” Gudschinsky's methodology could function as an initial heuristic by which potentially informative correspondences can be identified in comparative data. However, as a stand-alone procedure for inferring cognates, the methodology opens the door to a multitude of *ad hoc* explanations about conditioning environments; that is, it is not possible to independently test a hypothesis about a conditioning environment if it is relevant for only one set of sounds.

In adapting Gudschinsky's methodology to the historical study of sign languages, Woodward retained the notion of probable cognacy and its omission of the requirement for correspondences to regularly recur.

“Linguists working on lexicostatistics of sign languages should classify two forms as cognates using the same standards employed by linguists working on spoken languages, that is, only if the application of plausible rules can derive form A from form B, form B from form A, or both form A and form B from some other form that once existed or continues to exist in related languages. Such phonological rules can be rules of assimilation, dissimilation, deletion, epenthesis, coalescence, metathesis, maximal differentiation, centralization, and/or some other phonological process in sign languages recognized by modern linguistics” (Woodward, 2011, p. 41).

In traditional historical linguistics, the process outlined in Woodward's first two scenarios above—that is, the derivation of one contemporary sign from another contemporary sign—would be better described as borrowing from a related language because, by definition, cognate forms cannot be derived from sister languages. Rather, cognate forms in sister languages are derived from a form in a common ancestral language *via* linguistic descent, which is the situation described in Woodward's third scenario above.

If the cognate inference procedure allows for *ad hoc* accounts of conditioning environments, such as those allowed in Woodward's cognate inference procedure, there may be greater potential for the introduction of systematic bias—particularly when comparing sign languages that we believe to be related on extra-linguistic grounds. For example, because we know that Laurent Clerc was a signer of LSF, we may be more likely to formulate *ad hoc* explanations for differences across contemporary signs in ASL and LSF.

Despite the issue outlined above, one advantage to Woodward's approach is that it incorporates a model of historical sign change in the cognate inference procedure. As our understanding of language change among sign languages improves, our model of historical sign change could allow us to more accurately reconstruct the potential pathways along which signs may have historically developed.

#### Inferences Based on Measures of Phonetic Similarity

The second main approach to making inferences about the historical relations of signs bases these inferences on measures of phonetic similarity. In a lexical comparison of American, Australian, British, and New Zealand Sign Languages, McKee and Kennedy (2000) introduced an algorithmic methodology for inferring cognates. In their approach, the sign parameters of handshape, movement, location, and orientation were pairwise compared, with three mutually exclusive possible results: “identical,” in which all four parameters match; “related,” in which at least one of the parameters matches and at least one differs; and “different,” in which all of the parameters differ. Sign pairs in the identical and related categories were inferred to be cognates. Inferences about the historical relationships among the four languages in the study were based on the distribution of sign pairs across the three categories—identical, related, and different.

Because of its algorithmic nature, McKee and Kennedy's (2000) procedure for inferring cognates might potentially be viewed as more objective than Woodward's. Their approach also excludes one possibility for the introduction of systematic bias in historical comparisons of sign languages because their algorithm does not allow for *ad hoc* accounts of conditioning environments when parameter values differ (see the discussion of Woodward's approach in the previous section). However, McKee and Kennedy's approach places strict constraints on language change that may not have strong empirical or theoretical grounding. All four parameter values in a sign can change, including handshape (Battison et al., 1975), number of hands and movement (Frishberg, 1975), orientation (Wilcox and Wilcox, 1995), and location (Lucas et al., 2001; Schembri et al., 2009). But, for sign pairs to be inferred as cognates in McKee and Kennedy's approach, signs must have only minimally changed over time or they must have changed in exactly the same ways because all parameter values in a pairwise comparison must match for signs to be considered “identical,” and at least one parameter value must match for signs to be categorized as “related.”

As with Woodward's approach, McKee and Kennedy's methodology does not attempt to differentiate inherited vocabulary from borrowed vocabulary. Instead, it solely bases historical inferences on measures of phonetic similarity. That similarity could be due to inheritance, if two sign languages have inherited similar forms from a common ancestral language and those forms have not yet substantially changed. However, that similarity could also be due to borrowing or chance similarity. The inability of this methodology to differentiate vocabulary based on the differing processes by which that vocabulary has entered a language is a weakness that is inherent in any approach that bases historical inferences on phonetic similarity.

#### Recent Approaches to Historical Inferences

Relatively few sign language historical linguists in the twenty-first century have taken qualitative approaches in their historical comparisons. Supalla and Clark (2015; see Section Theoretical adaptation of the cognate) and Shaw and Delaporte's (2014) studies of the histories of signs in ASL are two notable exceptions to this observation. Many more historical comparative studies of sign languages have taken quantitative approaches, following Woodward and McKee and Kennedy (e.g., Parkhurst and Parkhurst, 2003; Sasaki, 2007). In sign language historical linguistics, this focus on quantitative approaches may ultimately stem from discussions within the field about the appropriateness of lexicostatistics for studying the histories of sign languages (Woodward, 2011). However, in historical linguistics more broadly there also has been a surge in the use of quantitative approaches over the past two decades. In that time, historical linguists have come to recognize how computational phylogenetic approaches and methods that developed in the fields of biology and systematics may help them to investigate questions about the historical evolution of languages and language families (Gray and Atkinson, 2003; Atkinson and Gray, 2005; Bouckaert et al., 2012; Kolipakam et al., 2018). Here I briefly highlight three recent studies that have used quantitative and computational phylogenetic approaches to compare signs and other linguistic features.

In a recent large-scale comparison of 23 sign languages, Yu et al. (2018) annotated signs based on Brentari's (1998) model of the sign and then computationally pairwise compared these annotations. Their comparison produced a distance matrix, which was used as the input for a hierarchical cluster analysis. Many of the clusters produced by their approach were expected based on our understanding of the extra-linguistic history of connections among signing communities. For example, LSF and *L*í*ngua Brasileira de Sinais* are closely grouped, as are *svenskt teckenspråk* and *L*í*ngua gestual portuguesa*. However, other clusters were unexpected: ASL was more closely grouped with Polski Język Migowy, Eesti viipekeel, and Latviešu zı̄mju valoda (Polish, Estonian, and Latvian Sign Languages) than with LSF; and Türk i°şaret Dili (Turkish Sign Language) was closely grouped with Íslenskt táknmál and Lingua dei Segni Italiana (Icelandic and Italian Sign Languages). Despite these unexpected results, Yu et al.'s study represented an innovative approach to studying the histories of sign languages; it is also one of the few available large-scale comparisons of sign languages. In a follow-up study, Abner et al. (2020) used a similar computational approach to study the distribution of phonological features across the languages in their sample and to make inferences about the historical development of sign language families based on the distribution of those features.

Power et al. (2020) designed a database of 76 manual alphabets, including those of contemporary sign languages and of historical manual alphabets dating to the sixteenth century. They compared handshapes in these manual alphabets by making qualitative judgements about the similarity of their forms. The manual alphabets were then pairwise compared and a series of computational phylogenetic network methods were applied to understand the complex patterns of similarity among these manual alphabets. Because the sample of manual alphabets included 36 historical examples, the authors were able to compare subsets of manual alphabets at various historical periods and to make inferences about their evolution over time. By assuming that the historical connections among manual alphabets paralleled historical connections among sign languages more broadly, the authors used their results to understand the world-wide dispersal of European sign languages.

In sum, recent work in sign language historical linguistics has followed broader trends in historical linguistics by applying computational and phylogenetic methods. Whereas previous quantitative comparisons mainly focused on sign vocabulary, the recent approaches highlighted here have studied other aspects of sign languages—such as their phonological features and manual alphabets—to better understand the histories of these languages. Thus, these recent approaches can also be viewed as alternative approaches to the Comparative Method. Like the previous approaches discussed in the preceding two sections, more recent approaches do not rigorously differentiate between inherited and borrowed signs or linguistic features.

### Etymological Relations

I have argued that one of the main problems that has shaped the theories and methods of sign language historical linguistics has been the inability to identify regular correspondences among apparently cognate signs. In this section, I briefly recapitulate that argument before discussing the notion of the etymological relation.

As I discussed in Section Relationships among spoken languages and among signed languages, the process of linguistic descent—that is, the native acquisition of language by children over multiple, successive generations—has been argued to be a driver of the type of incremental change that can result in regular correspondences (Labov, 2007). Because many sign languages that are thought to be related have not diversified *via* linguistic descent, we might not expect to find regular correspondences among the apparently cognate signs of these languages. If we cannot identify regular correspondences, we cannot use the Comparative Method to identify cognates or to rigorously differentiate inherited vocabulary from vocabulary that has entered a language due to other processes, such as borrowing. Given this problem situation, the term cognate is not, in my view, an appropriate characterization of the historical relations of many signs—perhaps even of similar signs in the languages of many sign language families. What is an appropriate characterization of the historical relations of these signs?

In his comparison of theoretical terminology in historical linguistics and evolutionary biology, List (2016) showed that some of this terminology does not map in similar ways onto abstract historical relations. For example, a fundamental notion in biological evolution is homology (attributed to Owen, 1843). According to List (2016, p. 120), “[h]omology is a very general historical relation between evolving objects. It does not specify the process from which the relation originated.” Homology is a superordinate concept describing “a relationship of common descent” (Koonin, 2005, p. 311), with three subtypes “based on the processes underlying the homology”—namely processes of speciation, gene duplication, and horizontal transmission (List, 2016, p. 120).

Homology is distinct from similarities arising through analogy—that is, the evolution of functionally-similar traits that have no specifically historical relation. One example of a process giving rise to analogy was the independent parallel evolution of wings in bats and birds, which did not arise from common historical pathways of descent; rather, wings independently evolved in birds and bats for functional reasons (Morrison et al., 2015). There are clear parallels in traditional historical linguistics to the distinction between homology and analogy. Greenberg's (1957) four causes of similarity differentiate between two causes that are thought to be historical—namely inheritance and borrowing—and two others that are considered nonhistorical—chance and sound symbolism. According to List (2016), however, there is no broadly accepted theoretical notion in historical linguistics that corresponds to the notion of homology. Theories in historical linguistics are certainly concerned with processes of language diversification *via* linguistic descent; they are also concerned with borrowing. But, historical linguists do not commonly make reference to an overarching term to describe both inherited and borrowed features.

In parallel to the concept of homology, List (2016) proposed the term *etymological relation* to encompass the historical relations of cognacy and borrowing (see also “sign language etymology,” Supalla and Clark, 2015). List's invocation of etymology seems appropriate as a parallel to homology because the concept has a long history in linguistics with precisely this meaning. Mailhammer (2015, p. 424) defined an etymology as “a historical account of the origin and the subsequent historical development of a linguistic item.” He distinguished between “internal” and “external,” or “contact,” etymologies. An internal etymology is one that describes the history of an inherited linguistic feature, whereas a contact etymology implicates borrowing events, or horizontal transmission. Mailhammer (2015, p. 432–433) pointed out that “the etymology of a linguistic item can comprise one or more cases of horizontal transmission” and that “a contact etymology necessarily combines internal and external etymologies, vertical and horizontal transmission.” Thus, in parallel to homologous biological traits, the linguist may speak of etymologically-related words, the histories of which connect at a shared common etymon.

List's notion of the etymological relation accurately captures the type of historical relation that the less precise notion of influence is intended to invoke in the theory of sign language relationships described in Section Theoretical adaptation of the cognate. Characterizing the historical relations of many signs as etymological directly acknowledges the methodological problems facing sign language historical linguistics—in particular the current inability to identify cognates using the Comparative Method. In contrast to previous theories about sign language relationships, the notion of etymology maintains important theoretical distinctions between vertical and horizontal pathways of descent in the histories of signs and linguistic features. A contact etymology, per Mailhammer, is flexible enough to incorporate instances of both vertical and horizontal transmission in the history of a sign, without committing historical linguists of sign languages to any conclusions about the genetic language relationships of the sign languages being compared.

## Conclusion

The two subfields of historical linguistics—namely, those focusing on spoken and signed languages—have rarely engaged one another, despite the relevance of both subfields to an overarching theory of language change. Why have they so rarely engaged with each other? As we have seen, the field of sign language historical linguistics since the 1970s has adopted many of the theories and methods that developed in traditional historical linguistics, including notions such as the language family and cognacy, as well as methods such as lexicostatistics. More recently, too, sign scholars have applied computational and phylogenetic methods in their historical comparisons of sign languages, thereby following broader trends in the approaches used in historical linguistics. Thus, in one sense, sign language historical linguists have indeed engaged with the theories and methods of spoken language historical linguistics.

However, I have argued that theoretical notions like the genetic language relationship, the language family based on genetic relationships, and cognacy do not straightforwardly map onto the processes of historical development that have characterized the diversification of many sign languages. In addition, the innovative methods that sign language historical linguists have developed as alternatives to the Comparative Method have both fostered progress in our understanding of the histories of sign languages and, perhaps, hindered cross-disciplinary engagement because these methods fundamentally differ from those used in traditional historical linguistics. Greater clarity about the strengths and weaknesses of our methods as well as their aims may foster greater collaboration in the future.

Much progress has been made in sign language historical linguistics since Stokoe, but, as I have argued here, fundamental theoretical and methodological problems remain. In my view, one of the main thrusts in future research in this area should be a concerted effort to identify regular correspondences among apparently related sign languages and across historical stages of the same sign language. To date, there have been few systematic attempts to do so (Power et al., 2019, 2021). Relatedly, there have been few systematic studies of diachronic change between different stages in the historical development of sign languages. For example, more than 40 years have passed since Frishberg's (1975) groundbreaking study of diachronic change in ASL, and few scholars have attempted to refine or to add to Frishberg's insights (see Shaw and Delaporte, 2014; Supalla and Clark, 2015). Another promising area for future research is the use of simulation studies to model the effects of differing processes of language transmission on language change (Gong et al., 2010; Gong and Shuai, 2016; Mudd et al., 2020) and to understand how iconicity may shape language change (Greenhill et al., 2009; Currie et al., 2010).

## Author Contributions

The author confirms being the sole contributor of this work and has approved it for publication.

## Funding

Support has come from the National Science Foundation grant BCS-1941560 Regularity and Genetic Relatedness in Sign Languages.

## Conflict of Interest

The author declares that the research was conducted in the absence of any commercial or financial relationships that could be construed as a potential conflict of interest.

## Publisher's Note

All claims expressed in this article are solely those of the authors and do not necessarily represent those of their affiliated organizations, or those of the publisher, the editors and the reviewers. Any product that may be evaluated in this article, or claim that may be made by its manufacturer, is not guaranteed or endorsed by the publisher.

## Footnotes

^1^Guerra Currie et al. (2002, p. 225) hypothesized that cultural ties between Spanish-speaking Mexico and Spain might “manifest themselves” in linguistic similarities among LSM and *Lengua de Signos Española*.

^2^Labov (2007) uses the term “transmission” in a restricted sense to mean the intergenerational transmission of language and its acquisition by children; see Section Linguistic descent and its consequences for theories of sign language relationships. Here I use the term in its more general sense.

^3^With hindsight, the modifier “normal” may have been ill-considered; see Thomason (2002, p. 102), who admits that the opposite notion of abnormality of transmission is “arguably pejorative.” More to the point, to my knowledge, there have been no careful empirical studies on the basis of which historical linguists might determine which are the normal, or perhaps the most common, pathways of transmission within the world's language communities.

^4^Schembri et al. (2010) point out that, perhaps unexpectedly, British Sign Language (BSL) has exerted only limited influence on many sign languages in countries that once formed part of the former British empire, such as India, Pakistan, and South Africa. For example, the authors report that there are relatively few signs in Indian Sign Language that may have origins in BSL—although the two-handed (BSL-origin) manual alphabet does appear to be in use among at least some Indian signers. In South Africa, they report that some schools—though certainly not all—may have used BSL as a medium of instruction (see Aarons and Akach, 1998 for a history of schools for the deaf in South Africa).
